# Pragmatics: Mapping Evidence on Enhancing Children’s Use of Linguistic and Non-Linguistic Capacities for Interactive Communication

**DOI:** 10.3390/children9091318

**Published:** 2022-08-29

**Authors:** Ahmed Alduais, Issa Al-Qaderi, Najla Alfadda, Hind Alfadda

**Affiliations:** 1 Department of Human Sciences, University of Verona, 37126 Verona, Italy; 2 Institute of English Studies, University of Warsaw, 01-445 Warsaw, Poland; 3 Department of English Language and Translation, King Saud University, Riyadh 11451, Saudi Arabia; 4 Department of Curriculum and Instruction, King Saud University, Riyadh 11362, Saudi Arabia

**Keywords:** pragmatics, interaction, linguistic competence, linguistic capacity, non-linguistic competence, communication disorders, scientometric review

## Abstract

New-born infants communicate from the first minute they come to life. This non-linguistic and non-verbal capacity to interact from the first day they come to life enables them to express their needs and evidence their typical development. This capacity to interact develops to include linguistic and non-linguistic use of verbal and non-verbal interaction, that is, pragmatics. Because pragmatics is heterogeneously structured of semiotic, cognitive, motor and sensory elements so it is vital to ensure successful human interaction. The other language elements (i.e., phonological, morphological, syntactic and semantic) are essential inputs for this human interaction outcome (i.e., pragmatics). Accordingly, this study sought to map evidence that pragmatics can enhance children’s use of linguistic and non-linguistic capacities for interactive communication. This was addressed by conducting bibliometric and scientometric analyses of 6554 documents from Scopus, 1167 from WOS and 11,230 from Lens between 1939 and 2022. We analysed the past, present and future developments of the field of pragmatics using bibliometric and scientometric indicators. The scientometric analysis was conducted using CiteSpace 5.8.R3 and VOSviewer 1.6.18 software, which enabled the tabulation, visualisation and measurement of the impact of central influencers in the field of pragmatics. In the light of our results, pragmatics continues to expand in order to understand human interaction in a deeper way and to enhance children’s typical interactions with the environment around them. The group should also include adults or elderly people whose pragmatic language skills have been impaired due to any acquired or developmental disorder, such as a brain injury.

## 1. Introduction

### 1.1. The Rise of Pragmatics

There has been tremendous growth in pragmatics over the years, from a lost field to perhaps the most expanding and interdisciplinary field in linguistics today. Initially, it encompassed philosophical considerations of the nature of human interaction, inferences of logical meaning based on communication and intended meaning and semiotic aspects of non-verbal communication [1,2,3,4,5,6,7,8,9]. The study of human interaction nature in pragmatics went beyond examining these aspects of human communication to explore and examine more interconnected aspects of it. As a result of the openness of this enquiry into pragmatics (i.e., human interaction), pragmatics has become a field of study including a number of sub-fields that are integrated with other sciences. Some of these emerging fields of pragmatics include historical pragmatics (i.e., the history of change in human interaction) [10], cognitive pragmatics (i.e., cognitive processes involved in human interaction) [11], neuropragmatics (localisation of brain areas and their functions concerning human interaction) [12], clinical pragmatics [13] (i.e., diagnosis, assessment, and rehabilitation of pragmatics in speech and communication disorders [14,15,16]) and proposedly psychopragmatics (i.e., acquisition and production of pragmatic language skills).

Before becoming an independent discipline of linguistics, pragmatics underwent three main phases. Charles Morris is considered the first philosopher who identified pragmatics in the 1930s [1]. Influenced by Charles Sanders Peirce’s work on the philosophy of pragmatism in 1905 [17], he is the first to identify pragmatics as one of the three subfields of the science of signs, namely syntax, semantics and pragmatics [1,5,18,19]. Morris argues that pragmatics is concerned with the study of the relationship between signs (i.e., linguistic expressions) and their users (as individuals and communities), as opposed to semiotics, which is only interested in the study of signs, or syntax, which is concerned with the study of the relationship of signs to each other, or semantics, which is the study of the relationship of signs to the things to which the signs are referring [1]. Morris considers pragmatics as a branch of semiotics and defines it as the study of the relation of signs to users [1], and to put differently, he refers to it as that branch of semiotics that deals with the origin, usage and signs’ effects [20].

During the 1960s and 1970s, pragmatics underwent a second phase of development. A pragmatic approach to language was considered incompatible with linguistic study during this period of time [19]. Philosophers of language such as Austin, Searle and Grice laid the foundations of pragmatics [21]. A new approach to the study of language emerged, viewing it as a type of human action [22]. With regard to Speech Act Theory, Austin’s 1962 book *How to Do Things with Words* made a significant contribution to pragmatics development [2]. John Searle, a student of Austin’s, contributed to the Speech Act Theory in 1969 and classified speech acts into five groups: representations, directives, commissives, expressives and explanations [3].

It is noteworthy that Paul Grice’s 1975 essay, Logic and Conversation, which described his theory of conversation in terms of the Cooperative Principle, associated conversational maxims, and the Implicature concept, made a significant contribution to pragmatics [2]. His theory sought to reflect the fundamental characteristics of verbal communication. Grice’s theory, in Thomas’ opinion, “attempts to explain how a hearer moves from what is said to what is meant, from the level of explicit meaning to the level of implicit meaning” [23] (p. 56). As Mey points out, the first theories of pragmatics were developed by philosophers of language, not linguists [18].

As a distinct discipline of linguistics, pragmatics started to take shape in the late 1970s and early 1980s (Thomas, 1995, as cited in) [21]. In his 1983 book *Principles of Pragmatics* [19]. Leech developed and elaborated the theoretical framework and foundational ideas of pragmatics. The International Pragmatics Association (IPrA) was established in 1986, and the publishing of The Journal of Pragmatics by Mey and Haberland in 1977 also occurred during this time period [24]. Since then, pragmatics has developed rapidly and is continuously studied by various experts. Several international conferences have also been organised on the topic, such as Viareggio in 1985, Antwerp in 1987, Barcelona in 1990, Kobe in 1993, Mexico in 1996, Reims in 1998 and Budapest in 2000 [18].

Since then, two schools of thought—Anglo-American and Continental—have emerged in pragmatics [23,24]. Pragmatics, according to the Anglo-American school, is the systematic study of meaning resulting from or reliant on the use of language [23]. Therefore, implicature, presupposition, speech acts, deixis and reference are among the main topics of pragmatics. This view is called the component view of pragmatics. According to this view, and to [23], a linguistic theory includes a number of key components, including phonetics, phonology, morphology, syntax and semantics. Thus, pragmatics is merely another essential element that contrasts with the language theory.

Similar to this, LoCastro demonstrates that pragmatics’ component view reflects its roots in the philosophy of language [24]. He contrasts this with the continental school, which incorporates a critical analysis of language use and links pragmatics to sociolinguistics and discourse analysis. In addition, according to Huang, in the continental European vision of linguistics, pragmatics is considered a functional perspective on all basic elements and “hyphen” areas of linguistics and beyond [23]. To put it another way, the Continental school emphasises empirical pragmatics and sociopragmatics, cross-cultural pragmatics and interpersonal pragmatics whereas the Anglo-American school concentrates on theoretical, philosophical and formal pragmatics.

Recently, pragmatics has gained prominence across several disciplines, including linguistics. According to Huang [23], pragmatics is one of the most active and rapidly expanding subfields of modern linguistics and the philosophy of language. In recent years, it has become increasingly popular in the fields of cognitive science, artificial intelligence, computer science, neurology, speech pathology and anthropology.

### 1.2. The Scope of Pragmatics

The meanings of pragmatics change depending on the context in which language is employed. The definitions that are frequently used, cited and referred to as the most influential interpretations of the field will be presented in this paper so that readers can gain a fairly thorough view on this topic.

Pragmatics has drawn the attention of many scholars and has been defined in various ways. The commonly oldest definition is by Charles Morris in 1938 [1] who defines pragmatics as “the relation of signs to their users” [25] (p. 361). In 1983 Leech contended that pragmatics should not only be defined from a philosophical perspective, but also from a linguistic one [19]. He put it another way by saying that “I shall redefine pragmatics for the purpose of linguistics, as the study of meaning in relation to speech situations” [19] (p. 6). Interestingly enough, Cohen defines pragmatics as follows: it is pragmatics that counts for why people “apologize” by using “Excuse me” in certain contexts and not using “sorry” [26]. In other words, pragmatics studies how communicative actions get meaning based on a particular context [27].

It should be noted that the definition of pragmatics places a strong emphasis on context [28,29]. Context can be either linguistic, such as the choice of words, or sociocultural, such as the relationship among participants [28]. For Crystal, 2003, as cited [30] (p. 2), context is all that concerns the users’ performance. Additionally, for Sperber and Wilson, the study of language use is referred to as pragmatics [31]. Other authors [32] further defines pragmatics as the study of language from the perspective of its users, particularly on the choices they make, the limitations they face while using language in social interactions, and the effects their language use has on people who are not participating in the communication process.

While Montague describes pragmatics as “a science that is concerned with indexical expressions” [33] (p. 1), Thomas defines pragmatics as the “meaning in use or meaning in context” [23] (p. 1). According to Katz and Fodor, “a theory that is related to the disambiguation of sentences by the contexts in which they were uttered” [34] is what pragmatics is (p. 177). The field of linguistics known as pragmatics is defined by Trask as the study of “how utterances communicate meaning in a context” [35]. (p. 226). He continues by saying that the fundamental principle of pragmatics is the disparity between what is said and what is meant [35]. According to Wierzbicka, pragmatics is defined as the discipline that studies verbal interaction between “I” and “you,” [36]. To help learners bridge the gap between sentence meaning and speaker’s meaning, pragmatics is defined as the study of how linguistic features and contextual elements interact in the interpretation of utterances [37].

According to Trosborg [38], pragmatics is a subfield of semiotics that examines the interaction between signs and their users. Pragmatics can be used to interpret what interlocutors mean in a specific situation and how the context affects what is stated, in addition to linguistic terms used in communication between interlocutors [9]. According to Levinson, pragmatics has little to do with linguistic structure. It only addresses rules for language usage and comprehension as well as context-dependent features of language structures [5]. In other words, pragmatics is the study of how language is utilised in relation to its environment [39]. As for Pütz and Neff-Aertselaer, the study of language used in social interactions to create interactive contexts is known as pragmatics [40]. In their subsequent argument, the authors state that “pragmatics as a usage-based perspective on the language sciences such as linguistics, the philosophy of language and sociology of language essentially focuses on the exploration of language used and the users of language in real-life situations and, more generally, on the principle which governs language in everyday interaction” [32] (p. ix).

Leech defines pragmatics as the study of meaning and its relation to speech situations [19]. Similar is the case with another definition made by Stalnaker; “pragmatics is the study of linguistic acts and the contexts in which they are performed” [33] (p. 34). Thomas sums up the definition of pragmatics in three words; “meaning in interaction” [23] (p. 22). The author points out that meaning is not something that is formed by the speaker or the hearer alone, nor is it something that is inherent in the words alone. Making sense is a dynamic process that involves the negotiation of meaning between the speaker and the listener as well as the utterance’s physical, social and linguistic contexts [23].

O’Keeffe et al. [34] define pragmatics as the study of the connection between context and meaning. Taking into account the steps that lead to a specific interpretation of a speech that is used in a specific context is what pragmatics is concerned with. Crystal defines pragmatics as the study of language from the perspective of its users, particularly with regard to the choices they make, the challenges they face while using language in social contact, and the impacts their language use has on the other party to a communication act [41].

Thomas criticises Crystal’s definition, saying that it ignores the importance of interaction and places too much emphasis on the message’s producer. Thomas claims that Crystal’s definition of pragmatics as speaker meaning ignores the hearer’s or utterance interpretation [23]. Yule, who defines pragmatics as “the study of meaning as communicated by a speaker (or writer) and interpreted by a listener (or reader)” [23] (p. 3), takes into account both of these points of view.

The following definition contains precise descriptions of pragmatics. First of all, pragmatics is defined by Yule [35] as “the study of speaker meaning”, or the study of what the speaker means and intends with his utterance. Second, pragmatics is “the study of contextual meaning”, which focuses on how context affects what is said and how speech is organised depending on who is listening. Third, pragmatics is the study of how more information is conveyed than is actually expressed. In other words, pragmatics is concerned with what inferences can be drawn from the speaker’s utterances. Finally, Yule emphasises that pragmatics is “the study of the expression of relative distance”, or how the perceived proximity of the speaker and hearer can influence what is said or not said. Yule [35] and Thomas [35] make the assumption that the notion of pragmatics includes both speaker meaning and utterance interpretation.

In a recent study, Mathias, Ardo and Jakonda define pragmatics as the study of meaning from the perspective of the language user [36]. Thomas offers a different definition of pragmatics as the study of “meaning in interaction” [35] (p. 22). Since language use is viewed as a dynamic process, pragmatics, in Thomas’ view, is meaning in interaction: both interlocutors are making meanings in communication and those meanings get influenced by the physical, social and linguistic context [35]. The study of human activities distinguishes pragmatics from other linguistic fields such as syntax and semantics. There are benefits and drawbacks to this analysis: on the one hand, studying the way people make sense of each other appears to be interesting but on the other hand, studying individuals and their minds seems to be quite challenging [35]. Furthermore, in theory, anything can be uttered, but in practice, communication is always constrained by certain social norms, according to Crystal [37].

Yule’s [9] definition of pragmatics as the study of speaker meaning, contextual meaning, how more gets communicated than is spoken, and the expression of relative distance leads to inferring the different aspects of pragmatics which were first introduced in the 1960s and 1970s by Austin [2] and Searle [3]. As the above definitions show, pragmatics covers a variety of aspects of interaction. The fields of interlanguage pragmatics and second language acquisition (SLA) frequently include and study the main components of pragmatics, which is concerned with examining language learners’ pragmatic competence.

### 1.3. Scientific Contributions for Pragmatics

Table 1 shows the 13 most significant source journals for pragmatic research along with a brief summary of the scope of each journal. The title of the source journal, the country in which the journal was published, the name of the publisher, the starting date, the number of volumes published to date and web addresses are also outlined.

### 1.4. Purpose of the Present Study

This study carries out a scientometric review, analysis and visualisation to achieve the set research objectives. It aims to provide academics and researchers with a comprehensive understanding of the field of pragmatics between 1939 and 2022 and the evolving issues in pragmatic studies with illustrative diagrams and maps. It is worth mentioning that in recent years, scientometric analysis is considered one of the most widely used research methods to evaluate the research development and performance of researchers, universities, countries and journals [38].

## 2. Methods

### 2.1. Research Methods

Scientometrics “analyses the quantitative aspects of the production, dissemination and use of scientific information with the aim of achieving a better understanding of the mechanisms of scientific research as a social activity” [39] (p. 6). Additionally, “scientometrics is a study of artifacts; one examines not science and scholarship but the products of those activities” [40] (p. 491). While many researchers believe that a scientometric review evaluates the quality of produced research, this is disputable as Egghe states that “the task of determining quality papers is especially difficult in BIS [bibliometrics, informetrics and scientometrics] due to the very heterogeneous origin of the researchers” [42] (p. 390). A major objective of scientometric studies is to “reveal characteristics of scientometric phenomena and processes in scientific research for more efficient management of science” [43] (p. 1). On this point, it is stated that “bibliometrics seems to have established itself as a reliable tool in the general assessment of research” [41] (p. 1).

A scientometric study uses indicators by type (e.g., publication, citation and reference) or form (e.g., quantitative, impact or impact of quantitative) to guide its conclusions [43]. In these studies, the concept of mapping knowledge domains is pivotal, which “creates an image that shows the development process and the structural relationship of scientific knowledge” [44] (p. 6205). These maps are “useful tools for tracking the frontiers of science and technology, facilitating knowledge management, and assisting scientific and technological decision-making” [44] (p. 6201). Research has shown that this method of study should be expanded to other areas in the humanities and social sciences, in addition to the pure, applied, medical and health sciences [45]. This paper explores the field of pragmatics and demonstrates the need for more sophisticated tools to facilitate the visualisation of data [46].

### 2.2. Measures

In scientometric studies, several types of indicators are utilised. A number of these indicators may be bibliometric by means of databases such as Scopus, Web of Science (WOS), Lens [47,48,49,50], or scientometric by means of analysis based on software [51]. Our study utilised both CiteSpace 5.8.R3 [52] and VOSviewer 1.6.18 [53].

In detail, these scientometric measures are bibliometric and scientometric. As a first step, we utilised the features available on Scopus, WOS and Lens to establish bibliometrics for the study of pragmatics. These data included publications by country, university, journal, research area, author, year and citation reports. WOS as well as Lens both provided more options for data retrieval, with the Lens having more flexibility.

The scientometric indicators differ from bibliometric indicators in that they do not concentrate on the size of production of a particular area (here pragmatics), but rather they emphasise the trend, direction and quality of knowledge produced. Following are the indicators we used from CiteSpace and VOSviewer. To evaluate the impact of the research, CiteSpace offers the evaluation of betweenness centrality, burst detection, co-citations, silhouette scores and sigma. In computing centrality, a path between two nodes is measured and is achieved when two nodes are located in close proximity [54]. Citation bursts are used to determine the frequency of an event within a specified period of time. For instance, the repeated citation of a reference over a period of time [55]. Co-citation occurs when two references are cited by a third reference [56]. When performing cluster analysis, silhouette scores are used to measure the consistency of each cluster with its related nodes [52]. Last but not the least, sigma evaluates the strength of a node on the basis of centrality, betweenness and citation burst [52]. The VOSviewer software allows the analysis of several scientometric factors. Our study considered citations with documents and authors and co-citations with publication source as units of analysis. Citations refer to citations of one another to determine relationships between them. We also examined cooccurrence which is determined by the occurrence of documents together when using keywords as the unit of analysis [53].

### 2.3. Data-Collection and Sample

For the retrieval of published studies in pragmatics, we used three databases: Scopus, WOS and Lens. We acknowledge that repetition is characteristic of all three databases, but we also considered the use of three databases to address the limitations of each. While there are several journals that appear both in Scopus and WOS, there are also journals that appear only in one of these databases [47,49,50]. Compared to these two, Lens is a more extensive database that includes a variety of data [48].

A search was conducted in the three databases on 9 March 2022. The search was restricted to English-language publications. As we reviewed all types of publications, we found many unrelated documents, including conference papers, proceedings and short communications. Initially, we attempted to search by topic, but a large number of results were retained. The team reviewed some random pages in the middle and end of the report, and found that so many of them were not consistent with our topic, pragmatics. Thus, the title, abstract and keywords were changed. As our purpose was to cover all publications listed in the three databases, we did not select a time limit. In all three databases this criterion was applied, except in Lens which did not display the option to restrict the search to a single word search “pragmatics” as was available in Scopus and the WOS. Therefore, we restricted our search for sources which included the word “pragmatics”. Table 2 lists the search strings used in the three databases.

### 2.4. Data Analysis

The data were exported from the three databases in different formats. As part of the bibliometric analysis, we saved the data as plain text, CSV and Excel files. Excel was used to convert the plain text and CSV files into the initial figures for the indicators of the development of pragmatics. Though Lens allows the use of all generated figures from their database as we did, we save the data for future use.

After exporting the data to CiteSpace and VOSviewer, the scientometric analysis was carried out. CiteSpace requires “plain text” format, so we exported this format from WOS, but for Scopus, we generated RIS and then converted it to plain text using the same software. In order to avoid duplicated documents, we ran the “deduplicate” feature for each database separately. The settings were all left at their defaults. This analysis was conducted twice, once for cited references and once for keywords. Tables were generated for summary information, citation burst tables and keywords burst tables. For VOSviewer, we generated three types of visualisations: network visualisation, overlay visualisation and density visualisation for each data of the three databases separately.

## 3. Results

### 3.1. Overview of Pragmatics Studies from Scopus, Web of Science, and Lens

**Figure 1:**
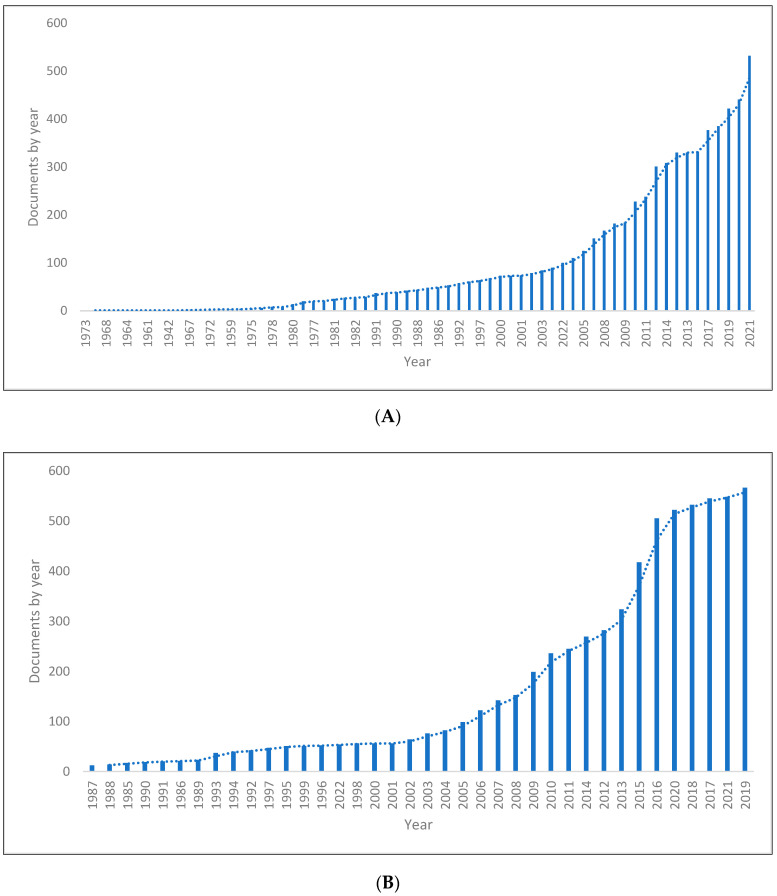

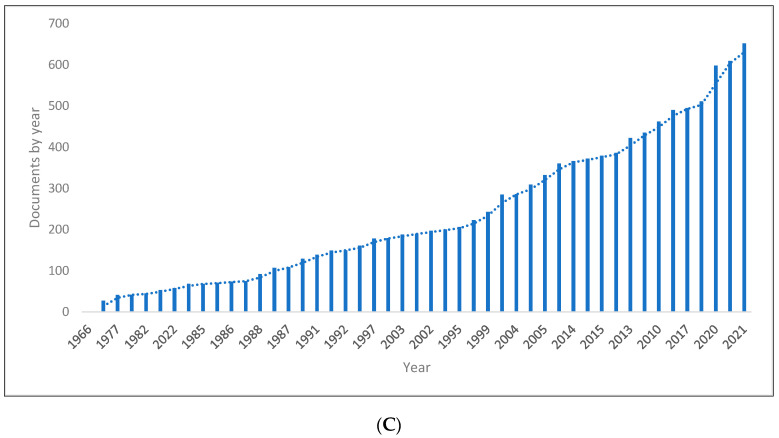
Pragmatics knowledge production size by year. (**A**) (Scopus). (**B**) (WOS). (**C**) (Lens).

A total of 6554 from Scopus, 6597 from the WOS, and 11,230 pragmatics papers were retrieved for analysis. The data periods for the three databases were 1939–2022, 1966–2022 and 1966–2022. Figure 1A–C shows the length of production by year for the three databases. It can be seen from the chart that there has been a significant rise in knowledge production in pragmatics since the last two decade, with the peak reached in the last years. Thus, these data demonstrate that pragmatic research has grown significantly over the past two decades. In other words, the data included 6554 documents from Scoups, of which 5480 were published between 2000 and 2022; 6597 from WOS, of which 6095 were published between 2000 and 2022; and 11,230 from Lens, of which 8561 were published between 2000 and 2022.

### 3.2. Production of Pragmatics Research by Country and University

**Figure 2:**
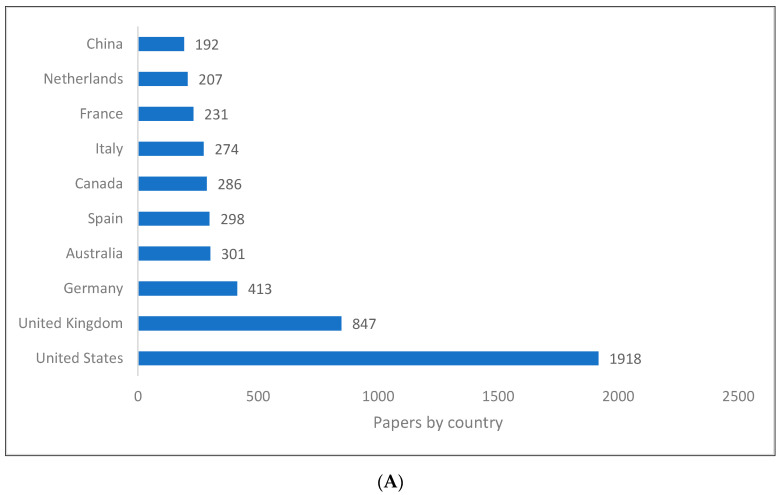

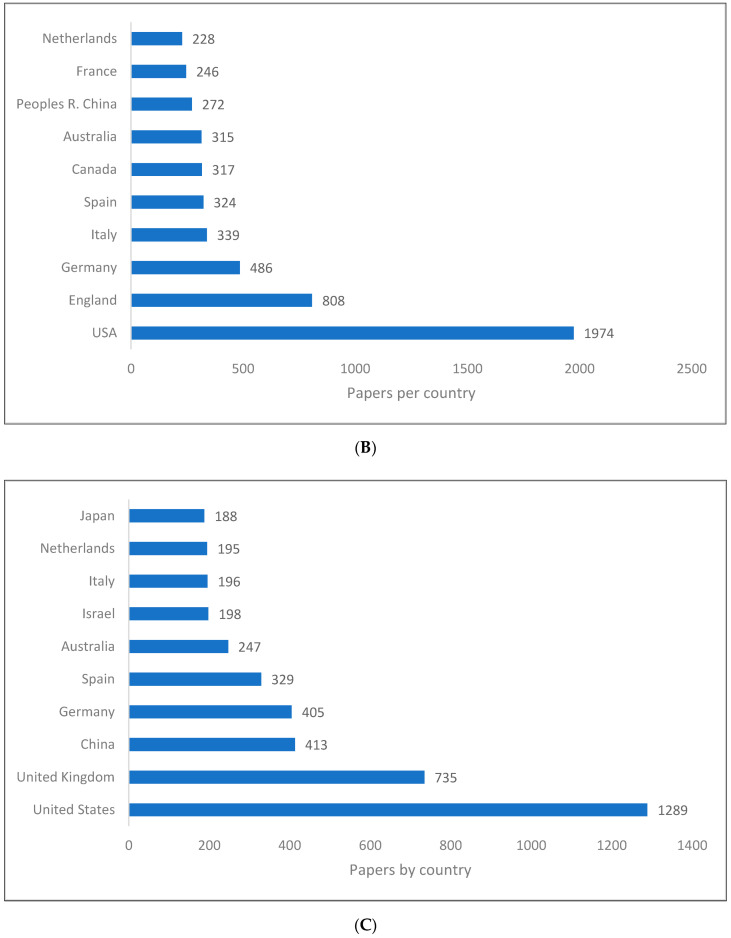
Pragmatics knowledge production size by country. (**A**) (Scopus). (**B**) (WOS). (**C**) (Lens).

It is evident that the ranking of the top producing countries for pragmatics knowledge varies depending on the type of database for several countries, but the USA and the UK remain the top two (Figure 2A–C). As an example, China ranked tenth on Scopus, eighth on the WOS and third on Lens. As another example, Iran appears only in the Lens database. Although Scopus and WOS include European and North American countries among the top 10 countries producing pragmatic research, Lens includes other countries (e.g., Japan, Israel). The reason for this could be attributed to the fact that Lens is a more comprehensive database that includes research that is not included in Scopus or the WOS.

**Figure 3:**
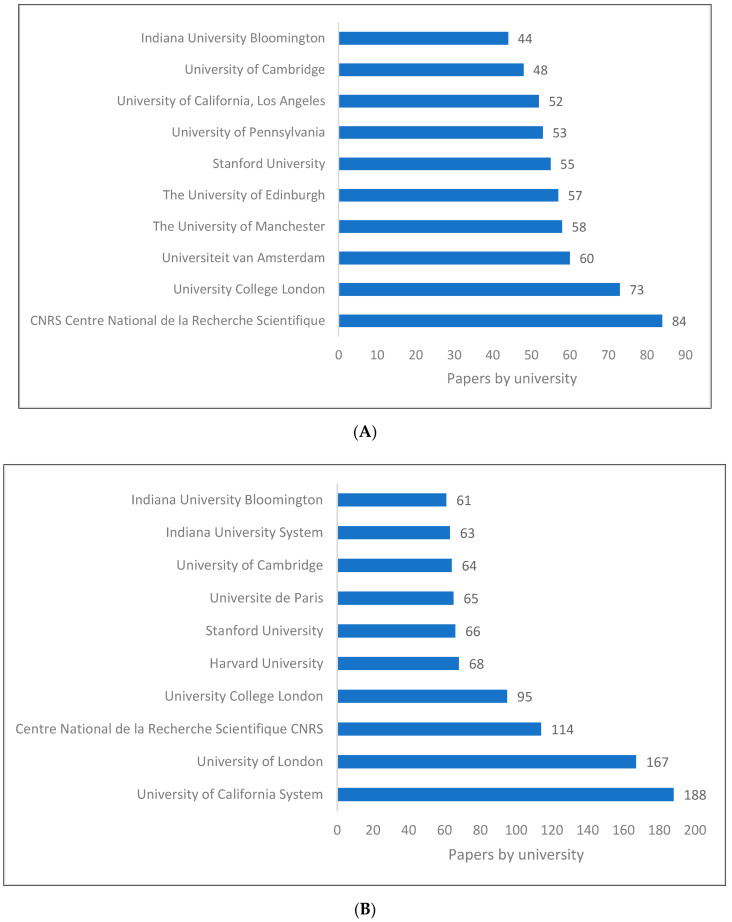

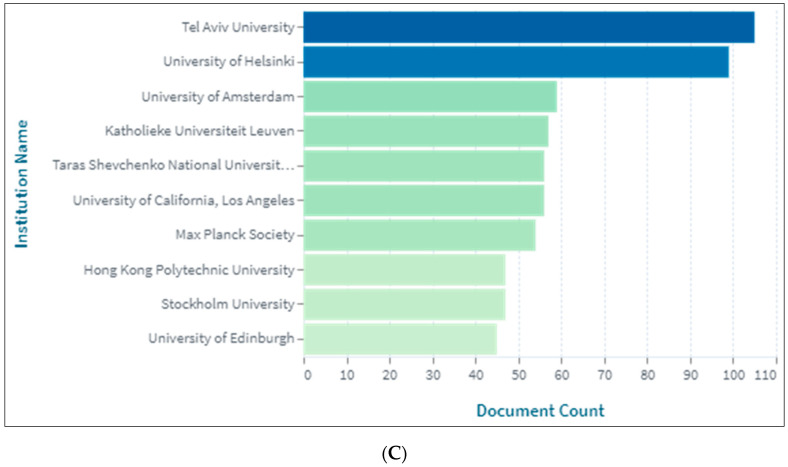
Pragmatics knowledge production size by university/research centre. (**A**) (Scopus). (**B**) (WOS). (**C**) (Lens).

Figure 3A–C shows the top universities and research centres producing research in pragmatics. The difference in ranking between the three databases can be attributed to the journals listed on each database. In all cases, the whole list is based in Europe and the United States, with a few exceptions appearing on the Lens list. There is more competition among US and UK universities, despite the fact that the top institution in Scopus is a French research institution. As opposed to this, the data retrieved from Lens shows that a university in Israel is the first on the list, followed by one in Finland, one in the Netherlands and one in Hong Kong SAR, China.

### 3.3. Production of Pragmatics Research by Journal and Publisher

**Figure 4:**
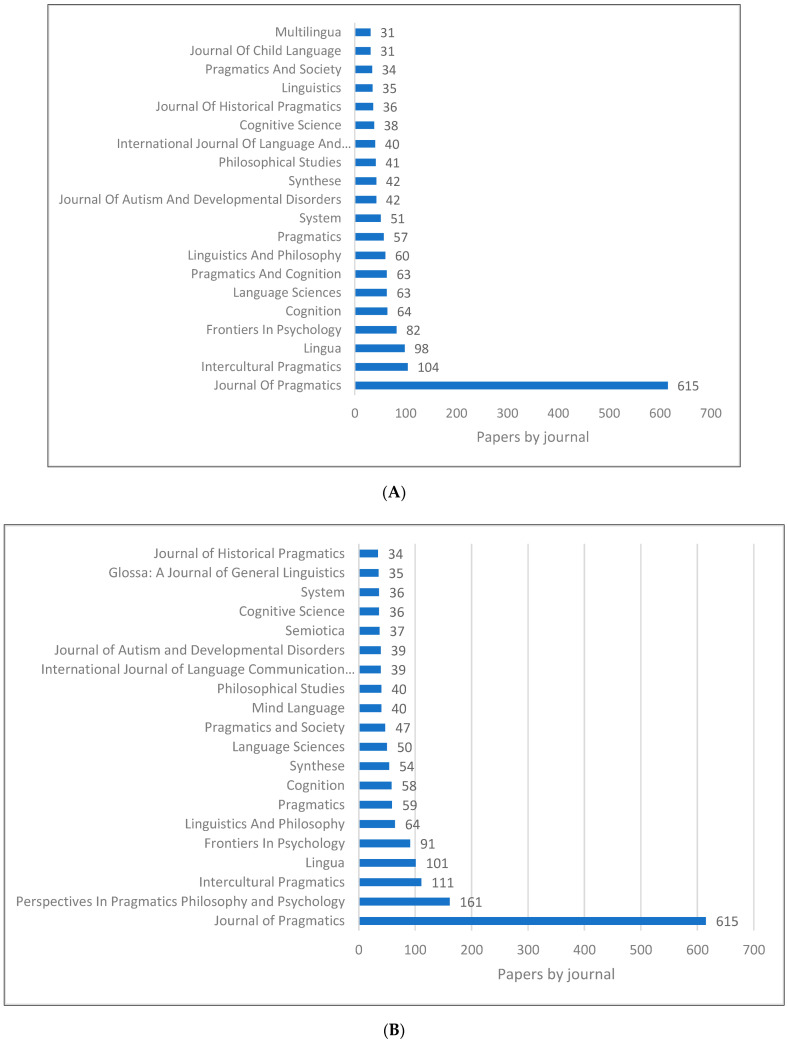

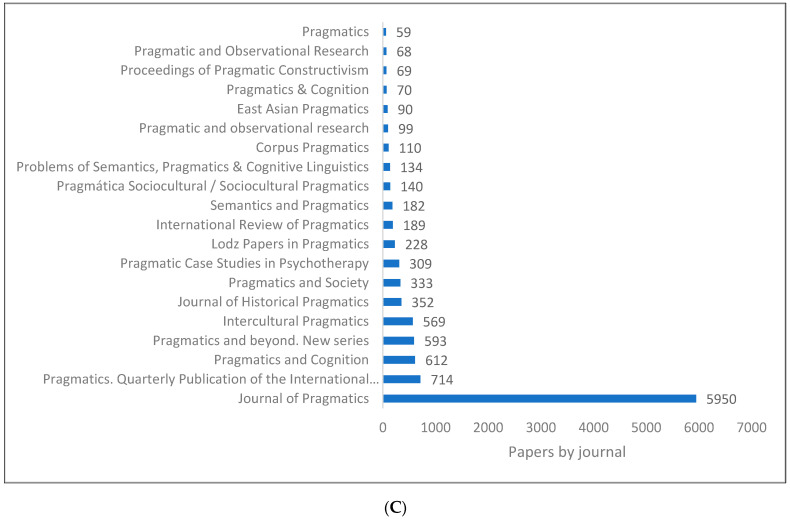
Pragmatics knowledge production size by journal. (**A**) (Scopus). (**B**) (WOS). (**C**) (Lens).

Figure 4A–C show the top 20 journals and book series publishing research in pragmatics. During the literature review, we have listed the journals that are specifically focused on pragmatics studies, which should be the top journals in this field. Nevertheless, despite the fact that the Journal of Pragmatics remains the top journal in all the databases, other journals vary greatly from one database to another. In the Scopus list, we can find journals such as Frontier in Psychology and Journal of Child Language. In these examples, pragmatics is integrated with educational psychology, developmental psychology and cognitive psychology. In addition to this, we can see the journal Cognitive Science. In this journal, topics related to the cognitive elements of pragmatics are published and discussed (e.g., executive functions, theory of mind).

**Figure 5:**
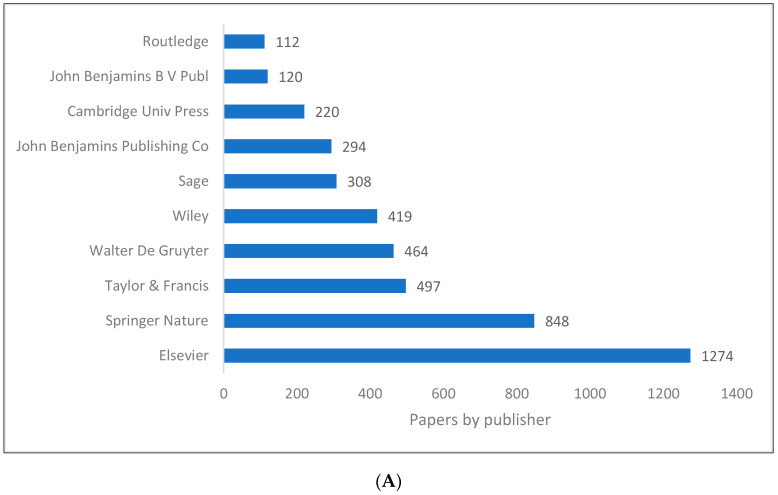

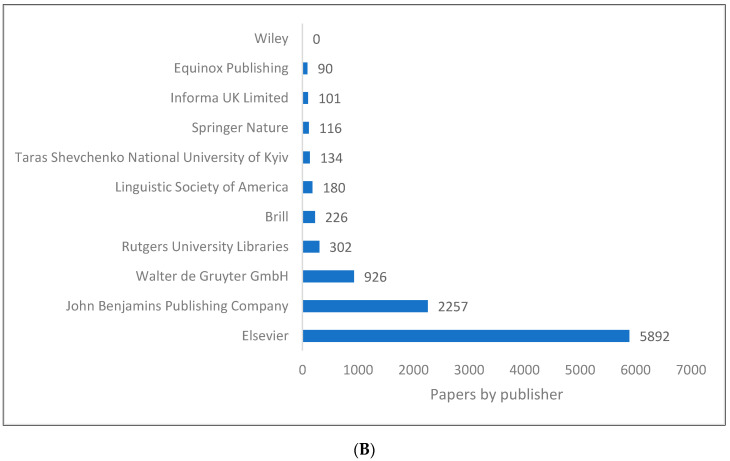
Pragmatics knowledge production size by publisher. (**A**) (WOS). (**B**) (Lens).

Figure 5A,B demonstrate the top publishers in pragmatics according to the data retrieved from the WOS and Lens. While Elsevier remains the leader in both databases, Springer Nature ranks second in the WOS, but eighth in Lens.

### 3.4. Production of Pragmatics by Research Area, Keywords and Cooccurrence

**Figure 6:**
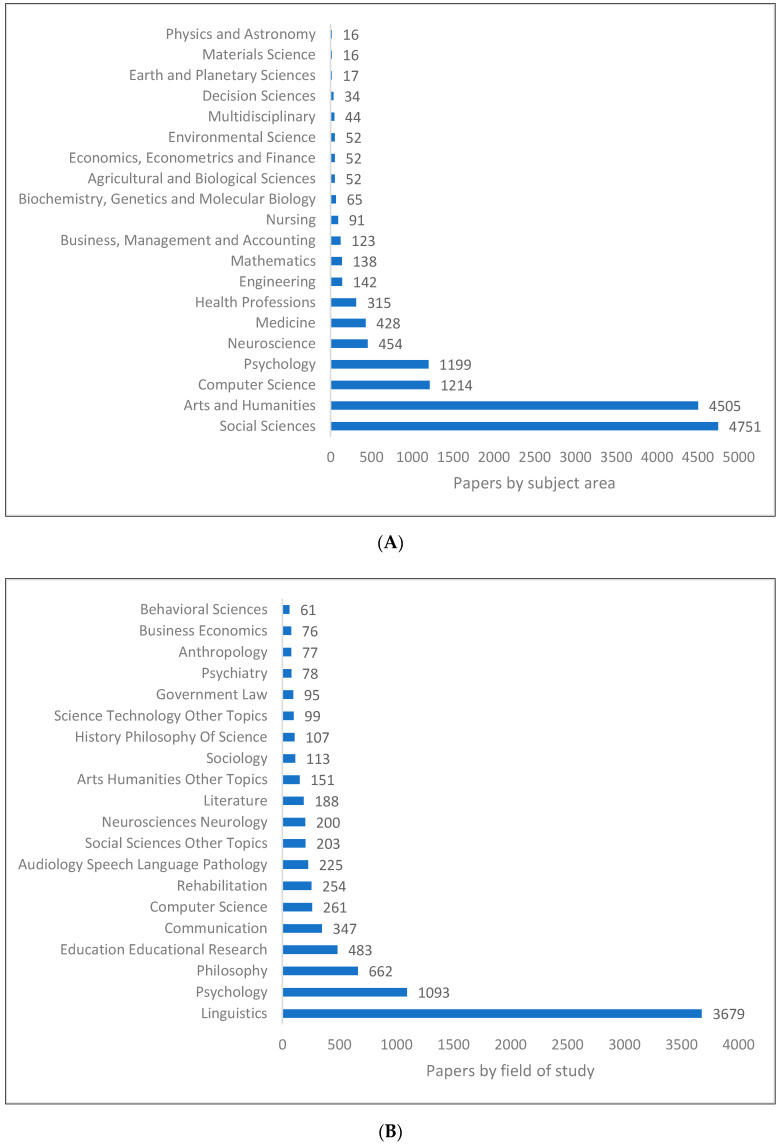

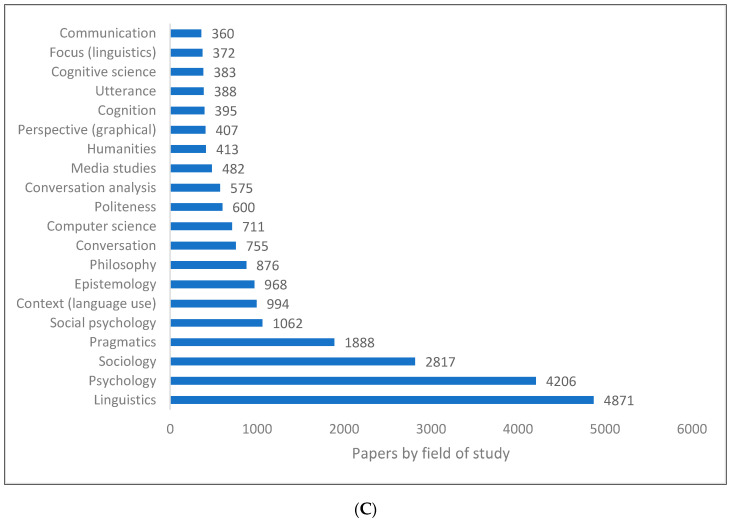
Pragmatics knowledge production size by research area. (**A**) (Scopus). (**B**) (WOS). (**C**) (Lens).

In spite of the fact that pragmatics is well-known as a field of study in linguistics, the study of pragmatics extends to other fields as shown in (Figure 6A–C). In Figure 6A, we see that social sciences, arts and humanities, computer sciences and psychology are the most common subject areas associated with pragmatics-related research. In Figure 6B, the top three fields of study related to pragmatics research are linguistics, psychology and philosophy. More specific fields are listed in Figure 6C, such as pragmatics, social psychology, language use, conversation, politeness and media studies.

**Figure 7:**
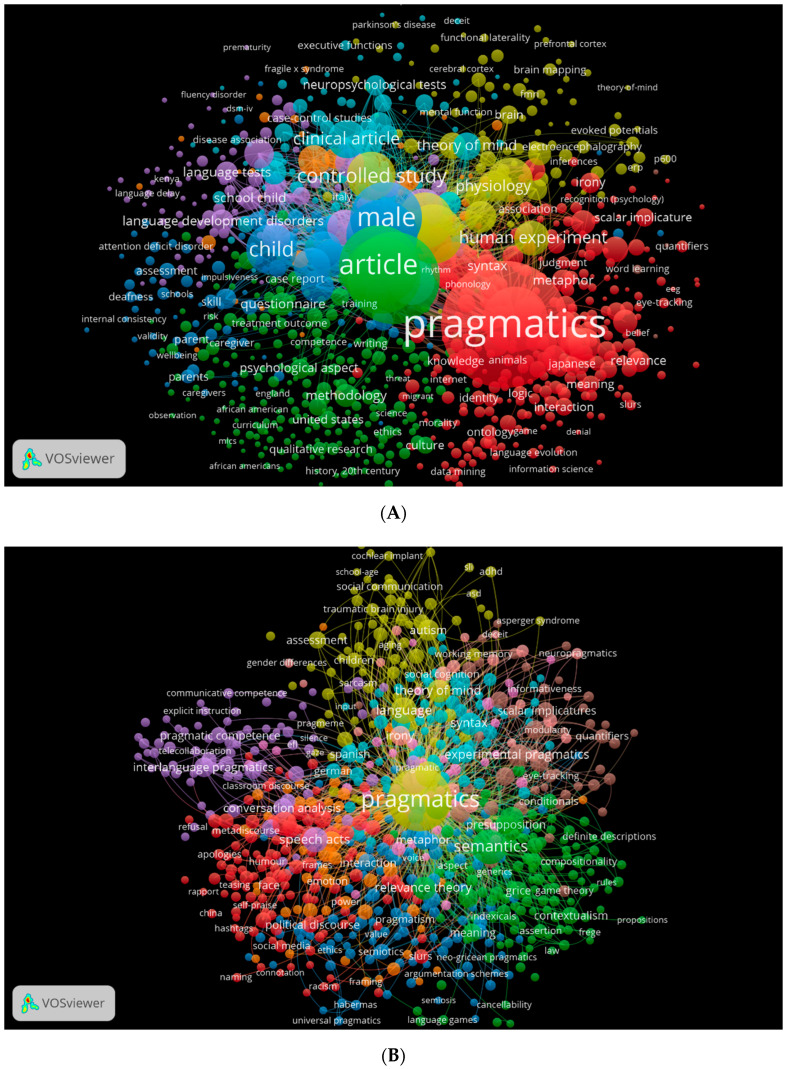

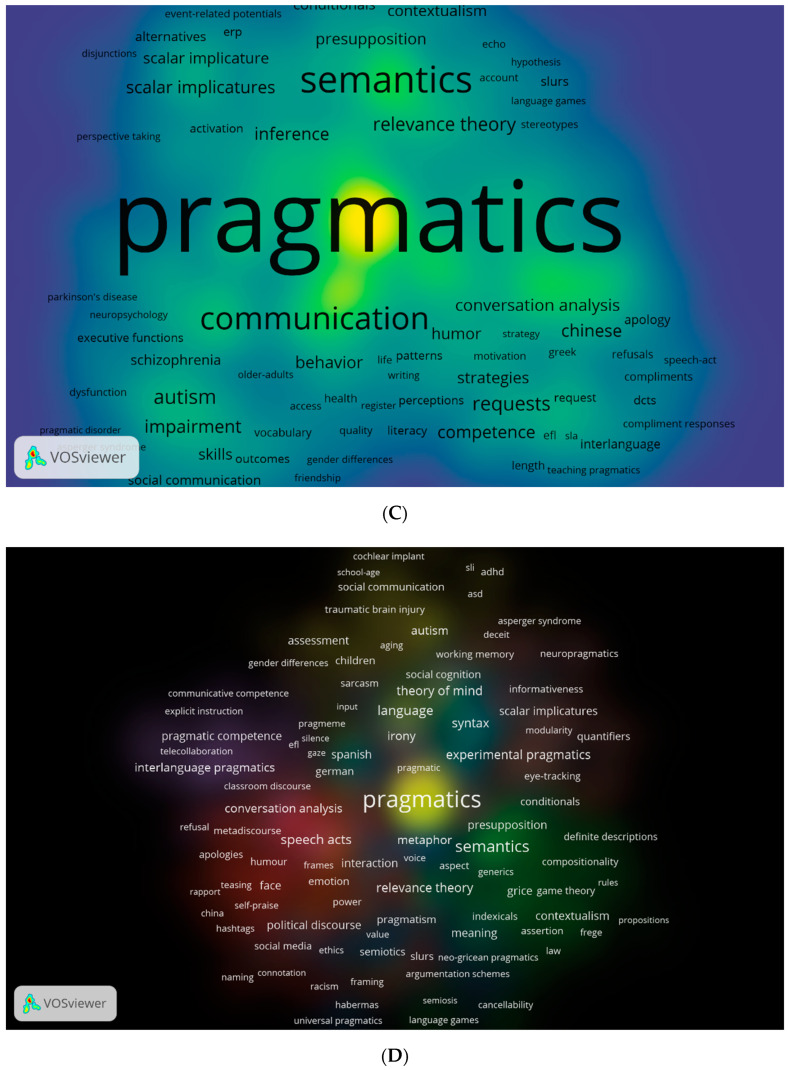
Cooccurrence by keyword network visualisation. (**A**) (Scopus). (**B**) (WOS) author keywords. (**C**) (WOS) cooccurrence by all keyword density visualisation. (**D**) (WOS) cooccurrence by author keyword density visualisation.

In addition to the research area and keywords, the cooccurrence of keywords is another important factor. Using VOSviewer, we generated three visual network maps showing the occurrence of keywords in pragmatics across the three databases (Figure 7A–D). Pragmatics can be studied in different directions, each represented by a different colour. The red colour indicates studies specifically focused on pragmatics within pragmatics itself. In yellow, studies appear to approach neuropragmatics and clinical pragmatics.

**Figure 8:**
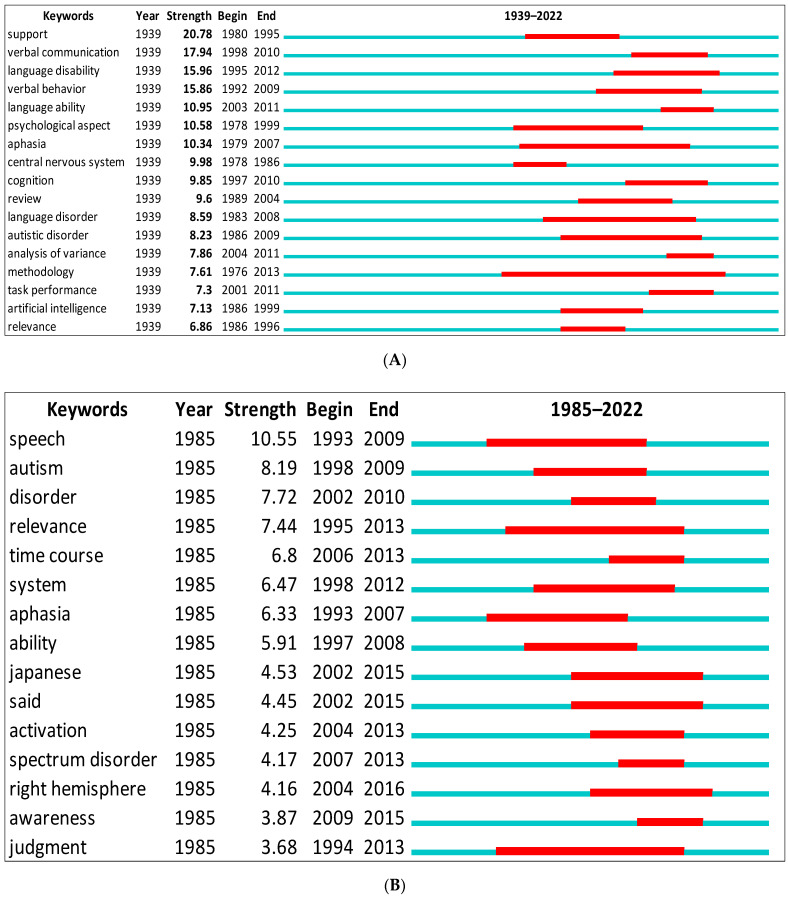
Top keywords with the strongest citation bursts. (**A**) Scopus. (**B**) WOS.

Additionally, we showed the top keywords with the strongest citation bursts from Scopus and WOS (Figure 8A,B). The green line indicates the period for all research. The red line indicates the beginning and end of the burst period. The word with the strongest citation burst in Scopus is (support = 20.78) between 1980 and 1995, and (speech = 10.55) between 1993 and 2009 for the WOS. The citation burst changes according to the database. For instance, while (relevance theory = 6.86) between 1986 and 1996 in Scopus, it has (7.44) between 1995 and 2013 in the WOS.

### 3.5. Production of Pragmatics by Authors

**Figure 9:**
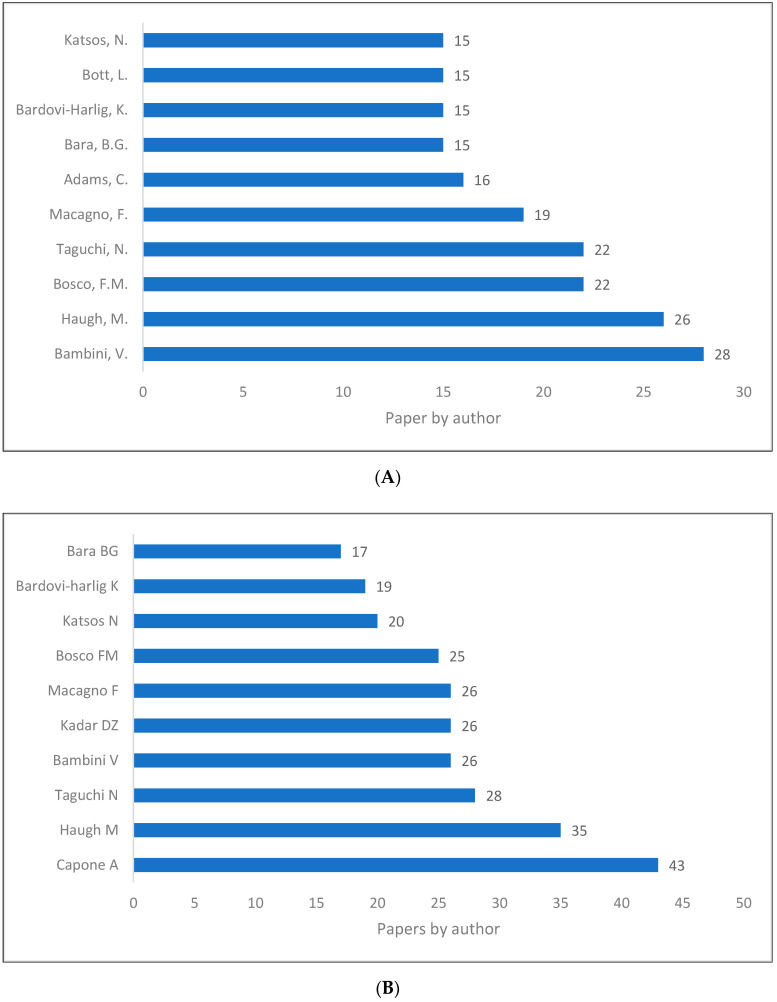

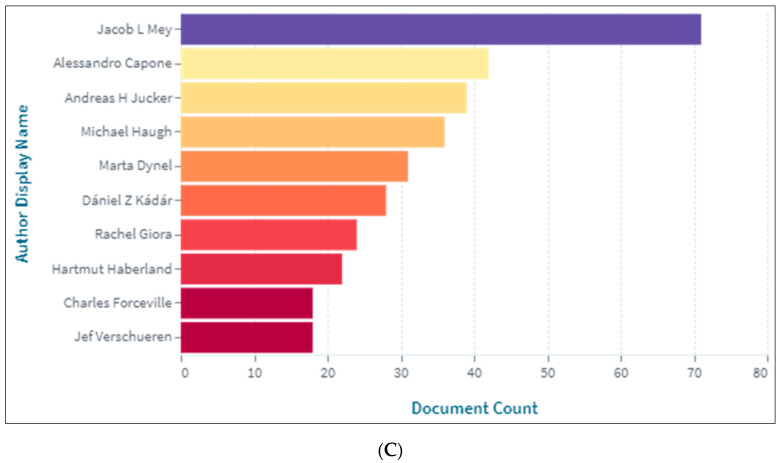
Pragmatics knowledge production size by author. (**A**) (Scopus). (**B**) (WOS). (**C**) (Lens).

There have been a number of authors who have made significant contributions to the field of pragmatics. We present the top ten authors according to the number of papers they have published as of 12 March 2022 (Figure 9A–C). It can be seen that there is a difference in the top authors among the databases. In Scopus, for example, the first author is Bambini [57], while in WOS, Capone [58] is the first author, and in Lens, Mey [18] is the first author.

### 3.6. Impact of Research on Pragmatics

**Figure 10:**
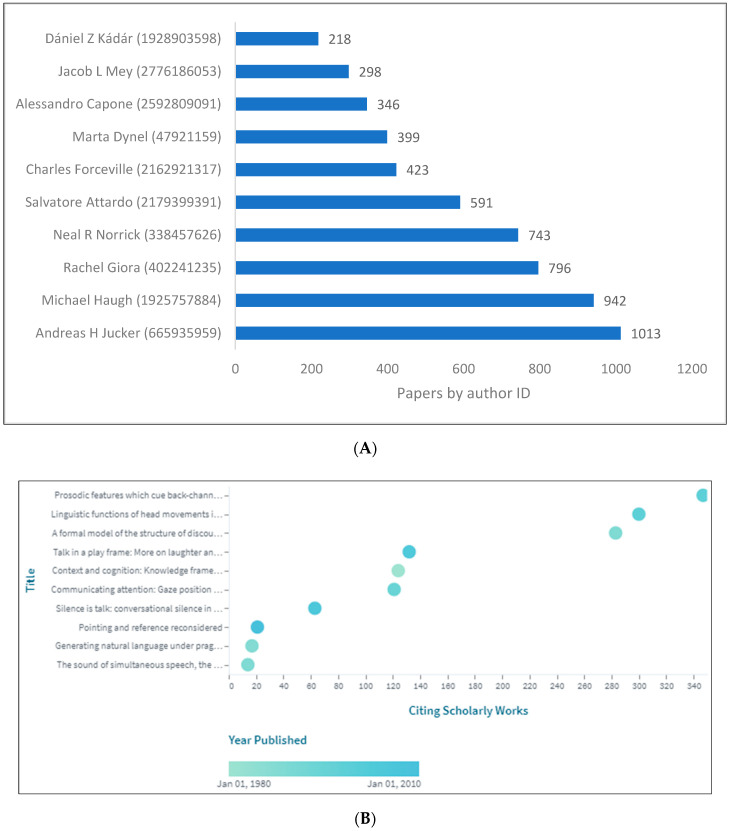
Pragmatics knowledge production size by author ID. (**A**) (Lens). (**B**) (Lens).

In order to measure the impact of produced research, we took into account a number of factors. We use the term impact when referring to the influence that authors have over other authors, and the extent to which their work is used by others. A number of factors were taken into account, including author ID by unique reference count from Lens database, citation reports from Scopus, and the WOS (Figure 10A,B) and (Table 3). Figure 10A,B show the top 10 authors and top cited works from Lens being referenced by authors according to their unique count referenced as compared to the top 10 being cited by others. There is a difference in ranking between the top 10 authors by number of publications. Jucker, for example, placed first in the Lens database. Table 3 shows the top 30 cited documents from Scopus and WOS. After merging them and removing duplicates, the results decreased to 25 documents. It has been found that the range of citations for Scopus is between 428 and 9320, 272 and 8781 for WOS, and 574 and 1927 for Lens.

**Figure 11:**
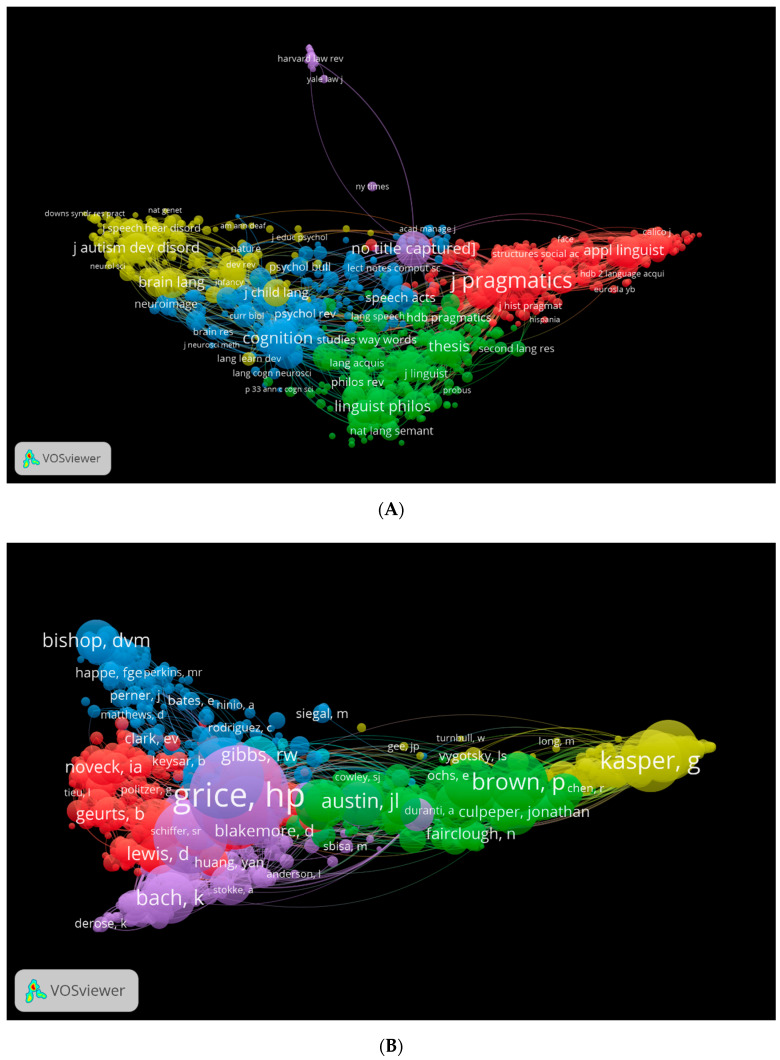

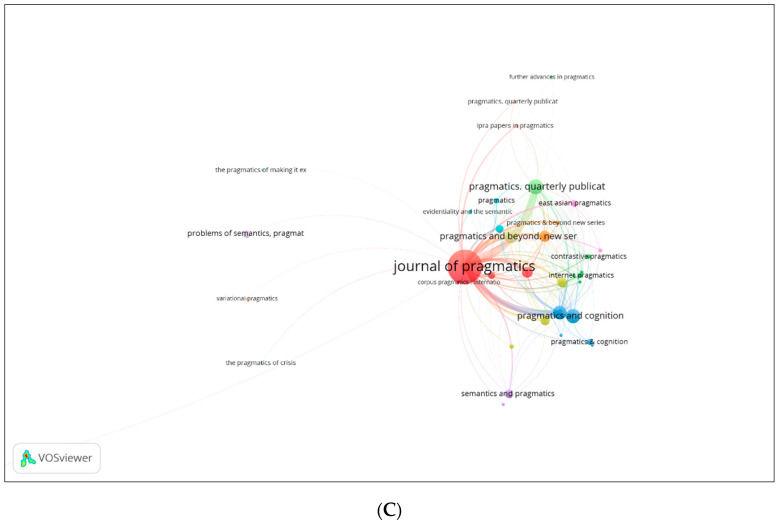
Co-citation by source, cited author network visualisation. (**A**) (WOS: source). (**B**) (WOS: cited authors). (**C**) (Lens: citation by source).

As a way to make this more tangible, we created three visual maps with the help of the VOSviewer (Figure 11A–C). An illustration of the co-citations by source is shown in Figure 11A. A different colour represents a different type of journal that publishes in the field of pragmatics. For instance, we see that all of the journals highlighted in red have a title or title phrase that involves the word “pragmatics”. There are a number of journals in blue that approach pragmatics in the context of cognition and psychological factors. The yellow journals are pertaining to a variety of topics in clinical pragmatics, neuropragmatics, speech and language disorders and other areas related to pragmatics. According to Figure 11B, Grice [84], Austin [85], Brown [86], Kasper [30] and Bishop [63] are the most cited authors. The authors of each of these works have contributed to pragmatics from a variety of perspectives. Grice [6], for example, is widely recognised as one of the most influential proponents of this field. Bishop [87], however, has published in pragmatics in relation to speech and language disorders. A citation map for journals according to Lens data is shown in Figure 11C.

### 3.7. Impact of Research on Pragmatics by Clusters, Citation Counts, Citation Bursts, Centrality, and Sigma

#### 3.7.1. Clusters

The network is divided into five co-citation clusters in the WOS data (see Table 4 for the remaining clusters.). The largest two clusters are summarised as follows. The largest cluster (#0) has 154 members and a silhouette value of 0.827. It is labelled as speech act by LLR, pragmatic competence by LSI and syntax-discourse interface (3.68) by MI. The most relevant citer to the cluster is Rieger [88], “How (not) to be rude: facilitating the acquisition of l2 (im)politeness”.

The network is divided into nine co-citation clusters in the Scopus data (see Table 4 for details). The largest three clusters are summarised as follows. The largest cluster (#0) has 418 members and a silhouette value of 0.663. It is labelled as autism spectrum disorder by LLR, natural language processing by LSI, and character equivalence (6.1) by MI. The most relevant citer to the cluster is Bambini [89], “Communication and pragmatic breakdowns in amyotrophic lateral sclerosis patients”.

#### 3.7.2. Citation Counts

**Figure 12:**
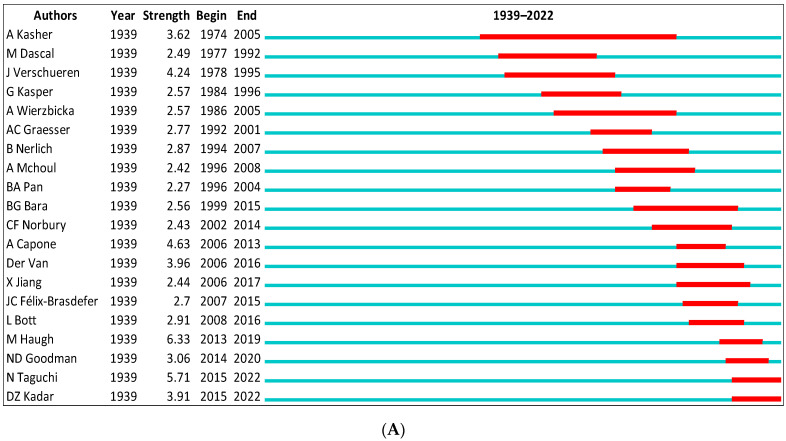

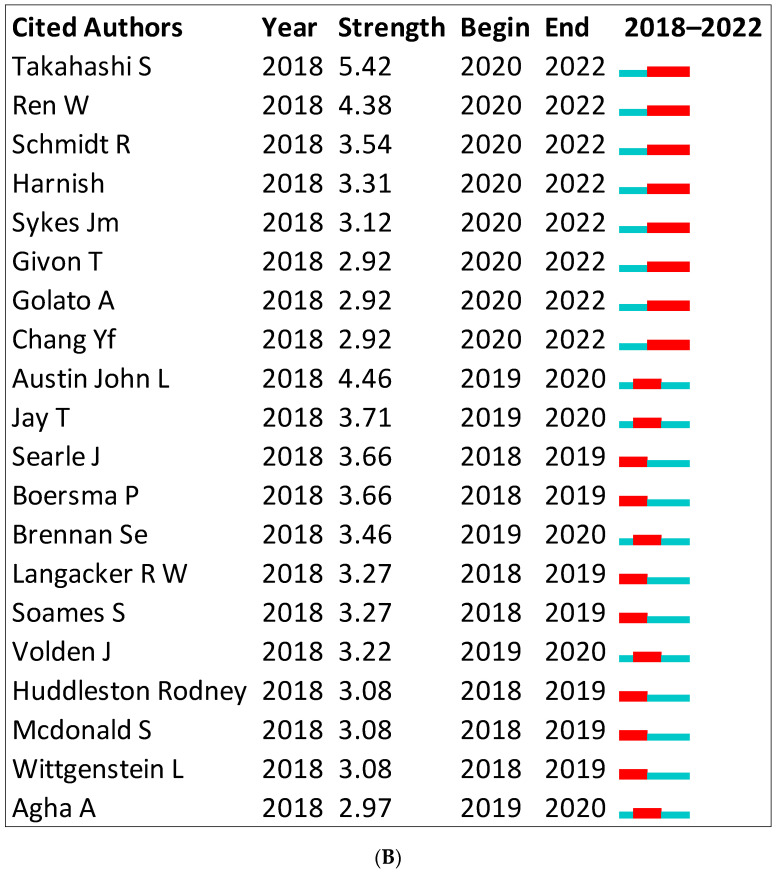
Top 20 authors with the strongest citation bursts. (**A**) (Scopus). (**B**) (WOS).

In the WOS, the top ranked item by citation counts is Grice [84] in Cluster #3, with citation counts of 231. In Scopus, the top ranked item by citation counts is Bambini [90] in Cluster #0, with citation counts of 25. The top 10 citation counts in Scopus and the WOS are listed in Table 5 and Figure 12A,B.

#### 3.7.3. Bursts

In the WOS data, the top ranked item by bursts is Brown [86] in Cluster #0, with bursts of 4.52. In Scopus, the top ranked item by bursts is Haugh [92] in Cluster #4, with bursts of 6.33. The top 10 bursts are listed in Table 6. 

#### 3.7.4. Centrality

In the WOS, the top ranked item by centrality is Grice [84] in Cluster #3, with centrality of 314. The second one is Brown [86] in Cluster #0, with centrality of 242. In Scopus, the top ranked item by centrality is Bambini [90] in Cluster #0, with centrality of 14. The second one is Der Van [91] in Cluster #1, with centrality of 12. The top ten central authors in pragmatics are listed in Table 7.

#### 3.7.5. Sigma

In the WOS, the top ranked item by sigma is Grice [84] in Cluster #3, with sigma of 0.00. The second one is Brown [86] in Cluster #0, with sigma of 0.00. In Scopus, the top ranked item by sigma is Bambini [90] in Cluster #0, with sigma of 0.00. The second one is Der Van [91] in Cluster #1, with sigma of 0.00. See Table 8 for the top 10 sigma values for authors in pragmatics.

## 4. Discussion

With the aid of visualising images and applying scientometric analysis to pragmatics research, the study aimed to provide academic researchers with a comprehensive presentation of pragmatics in its various facets and to provide them with an overview of how pragmatics is evolving and what research in the field requires. Both bibliometric and scientometric indicators were presented in relation to the field of pragmatics. Seven bibliometric indicators utilised the available features on Scopus, WOS and Lens. These were: publication by country, university, journal, research area, author, year and citation reports. Scientometrics indicators, on the other hand, included indicators used from CiteSpace and VOSviewer such as citation, co-citation and cooccurrence indicators.

Results indicate that: (1) The production of knowledge in pragmatics increased in the last two decades; (2) the top two producing countries are the USA and UK; (3) universities in the UK and the USA are the top in producing research in pragmatic; (4) the *Journal of Pragmatics* is the top in producing research on the field of pragmatics; (5) linguistics, psychology, and philosophy are the three top fields tackling issues associated with pragmatics; (6) Bambini [57], Capone [58], and Mey [18] are the top three authors on papers published till 12 March 2022; (7) finally, Elsevier and Springer Nature are the top contributors to the field. Five other results from this study merit comment.

To begin with, as we demonstrated in the results section, pragmatics research has seen a significant increase over the period 2000 to 2022 compared to previous years. Therefore, it was evident from 24,381 pragmatics documents published between 1939 and 2022, of which 20,136 were published between 2000 and 2021. As a result of linking this with the scientometric indicators, we can also identify the most searched and/or (co)occurring keywords researchers use when searching or conducting research in pragmatics. These included support [117], verbal communication [118], language disability [119], verbal behaviour [120] and language ability [121]. Most of these studies dealt with pragmatics from a conversational point of view through which pragmatic strategies as implicature are used to support communication and enhance foreign language communication (e.g., Hay’s offering sympathy to support humour) [117]. Another list included speech [122], autism [123], disorder [124] and time course [125]. Such studies stress the idea that pragmatic competence is not a homogeneous action but requires competence in a variety of comprehension tasks such as coherence and interpreting metaphors.

Second, we discussed in the introduction and results how pragmatics is integrated with various fields of study. We also underscored that pragmatics has evolved into a field that includes other subfields (e.g., neuropragmatics, clinical pragmatics). Since we analysed a large set of data involving over 24,000 documents, no prior assumptions were made regarding the nature of relationships between them except for the fact that they all used the concept of “pragmatics”. Having said that, cluster analysis enabled us to group this vast amount of data into associated patterns. These included natural language processing [120] and autism spectrum disorder [123]. On the latter, the largest clusters are pragmatic competence [126] and case study [127]. Pragmatics as part of language competence, especially in the past few decades, attracted the attention of linguists as an essential part of language competence and a necessary component in target language acquisition. When looking at these clusters more closely, they can be classified into two patterns. The first groups of patterns focused on examining syntax-discourse interface in relation to speech acts and pragmatic competence, and conducing case studies from the relevance theory perspective. The second group of patterns examined character equivalence in relation to autism spectrum disorders, natural language processing, pragmatic language and pragmatic disorder.

Third, it may be illogical to limit the contribution of authors to pragmatic contributions, but it is beneficial to highlight those who have contributed the most. The identification of the most influential pragmatics contributors reflects their deep understanding of the field. As a result of their focus on conducting research in this area, they may have more insightful findings than others. This led us to identify the top authors in the field of pragmatics, which included Bambini [57], Haugh [128], Bosco [11], Taguchi [129] and Capone [58]. Empirical approaches are introduced to pragmatics such as neuropragmatics [57] to be the brain activity involved in the pragmatic level of communication. Those researchers also stress the complexity of the situation that can include inferential work underlying communication and discourse.

The fourth point is that we are in support of the idea that classic and secondary sources may be found in any field. The former refers to the works authored by proponents of the fields (e.g., pragmatics), while the latter refers to research conducted after the authorship of such classic sources. In spite of this, researchers should be cautioned not to blindly cite these classic documents. Scientometric researchers refer to these documents as the top cited articles. As part of this study, we also identified the most cited pragmatics documents. These included the discussion of different topic of which are qualitative content analysis in nursing research [74], pedagogy of multiliteracies [60], measuring recognition [130] and the pragmatics of model-driven developments [79]. Such studies provide measures to achieve trustworthiness in qualitative content analysis including credibility, dependability, and transferability. They also utilise the importance of multiliteracies approaches to pedagogy to achieve learning.

Fifth, in addition to identifying the most cited authors, it is also possible to identify the authors who are likely to receive an increased amount of attention from researchers in the field of pragmatics. One possible explanation is that such authors have examined some emerging or controversial topics in pragmatics, which results in their research being cited more frequently. Accordingly, we used the Sigma metrics analysis to identify the top ranked researchers who are likely to receive a significant growth in citations. These included topics such as speech acts [131] and politeness and pragmatics [86]. Pioneering concepts in pragmatics are introduced as conversational implicature, politeness theory, and speech acts. Among the authors are Bambini [90] who outlines a new model of communication incorporating neurofunctional accounts of language with recent advances in neuropragmatics and van der Auwera [91] who, in a number of studies, contributes a comprehensive survey of the literature in conditional perfection.

Recent research in pragmatics supports the above findings. It is possible to find a reflection of these results in the most recent publications in pragmatics between 2021 and 2022 if we examine the most recent publications in pragmatics between these two years. Research in recent years has focused on L2 pragmatic comprehension [132], speech acts in virtual reality [133], pragmatic disorder in schizophrenia [134], request production by EFL learners [135], and role of theory of mind in physical and mental metaphors [136]. Although these five studies share the idea that they use pragmatics in their research, they approach it from different perspectives. For instance, we can see discussing pragmatic in relation to L2 and EFL learners in [135,137] (i.e., cultural pragmatics), pragmatics and mental health [134] (i.e., clinical pragmatics), and processing of pragmatics elements [136] (i.e., psychpragmatics).

It is evident in most existing research in pragmatics that there is an expanding trend of pragmatics research and a heterogeneous nature of the researchers in this field. Recent studies have examined hedging strategies in financial communication settings [138], linguistic vs. non-linguistic pragmatics [139], acquisition of presuppositional skills by preschoolers [140], expressing surprise and disapproval [141], conditionals in semantic-pragmatic interface [142], use of technology [143] or interactional pragmatic resources [144] to enhance L2 learning of pragmatics and assessment diagnosis of pragmatic language impairment [14,15].

Research in pragmatics has also been demonstrated to correlate with the clusters identified in our scientometric analysis. Among these studies are those investigating variations in pragmatic language learning in non-English contexts [145], aspects of politeness [146], using virtual reality for developing pragmatic language [147], pragmatics and reasoning [148], ritual interaction [132], hearing impairment and pragmatic language skills [149], autism and neural association of pragmatic language skills [150], linguistic prediction at the pragmatic language level [151], mind reading in pragmatics [152,153] and executive functions and pragmatic language skills [154].

### 4.1. Theoretical Implications

Scientists should apply caution in the interpretation of the results of scientometric studies [155] this is true regardless of the fact that these types of studies are becoming more popular in the present day [156,157]. In order to accomplish this, the data must be retrieved from a variety of sources, and it should not be limited to one database except for very well-founded reasons (that is, in this study we used Scopus, WOS and Lens databases). It would be helpful to use different tools for the analysis of the data in order to get the ability to incorporate various scientometric indicators at the same time (i.e., in this study we used both CiteSpace and VOSviewer).

An additional theoretical implication relates to the expansion of pragmatics into interdisciplinary fields. While this expansion is necessary to reflect the heterogeneous nature of pragmatic elements, it may lead to a deviation from pragmatic scope. As a result, it may result in divergence among researchers who are trying to reach new findings or associations related to human interactions. The need exists for the establishment of interdisciplinary research centres to examine human interaction (pragmatics) from a variety of perspectives, using a variety of methodologies and disciplines.

### 4.2. Practical Implications

Several practical implications can be drawn from this study. First, CiteSpace probably *failed* to analyse large data sets when they were set for references and cited authors. For other features (e.g., keywords, authors) this was possible. This resulted in the retrieval of a small amount of data over 2000 documents. While this step did not affect the results of showing the most cited references and clusters, it is a limitation of this software. After running the analysis for one full day, it did not even replicate the exact problem. Only after the data was decreased was this problem resolved. The second implication pertains to the three databases we used. When you have large data sets, the WOS only permits the export of 500 references at a time. There is a limit of 2000 references with full citation information in Scopus. In spite of the fact that we were able to divide the data by date and export it all, the process was time consuming. A limitation of Lens is that it does not allow precise searches when the keyword is a single word (for example, pragmatics).

### 4.3. Limitations and Future Directions

Certain limitations of this study could be addressed in future research. For example, the search strings used in the scientometric analysis were limited to the concept “pragmatics”. Despite the fact that we performed this step in order to reduce the amount of data retrieved and match the software requirements for smooth analysis, adding more concepts, themes or areas in pragmatics, would likely lead to a different direction in the research of pragmatics in terms of both bibliometric and scientometric indicators used in this study. The analysis of clusters is another example. Over 24,000 pragmatics documents were analysed and grouped into different clusters. Nonetheless, we did not examine these associated patterns of pragmatics and their meaning in relation to current research trends in pragmatics. The next step could be to conduct a cluster analysis to examine in detail such emerging patterns. 

## 5. Conclusions

This study aims to gain a better understanding of the rise and development of pragmatics. A total of 24,381 documents in pragmatics were analysed from Scopus, WOS and Lens between 1939 and 2022. The scientometric analysis of the development of pragmatics was performed using CiteSpace and VOSviewer. We have tabulated and visualised the size of the knowledge production in pragmatics and the central authors, topics, themes and documents that are directing research in this area of study. For instance, the UK and the US are among the top countries producing knowledge in pragmatics. Additionally, pragmatics is integrated with several fields of study, including linguistics, speech-language pathology, anthropology, sociolinguistics, cognitive sciences, neuroscience, forensic science, etc. Consequently, pragmatics has become a central field of study in the study of communication skills, cultural differences in communication, measuring verbal and non-verbal communication as well as measuring cognitive abilities in several types of developmental and acquired disorders.

## Figures and Tables

**Table 1:** Top 13 Journals Exclusive to Pragmatics Research. (all URL accessed on 10 February 2022).

Source Journal	Host Country	Publisher	Span	Volumes	Web Address	Scope of the Journal
Journal of Pragmatics	Netherlands	Elsevier	1977–2020	191	https://www.journals.elsevier.com/journal-of-pragmatics	Innovative pragmatic studies from all perspectives.
Intercultural Pragmatics	Germany	De Gruyter Mouton	2004–2020	18	https://www.journals.elsevier.com/journal-of-pragmatics	General theoretic issues, multiple languages and cultures, or various varieties of a language.
Pragmatics and Cognition	Netherlands	John Benjamins Publishing Company	1993–2014, 2016–2019	27	https://benjamins.com/catalog/pc	Linguistics, semiotics, cognitive science, neuroscience, artificial intelligence, philosophy, ethology and cognitive anthropology.
Pragmatics	Belgium	International Pragmatics Association	1986–2020	31	https://benjamins.com/catalog/prag	Linguistics, anthropology, sociology, psychology or computation.
Journal of Historical Pragmatics	Netherlands	John Benjamins Publishing Company	2000–2019	22	https://benjamins.com/catalog/jhp	Socio-historical and pragmatic aspects of historical texts within the context of socio-cultural communication as well as diachronic pragmatics.
Pragmatics and Society	Netherlands	John Benjamins Publishing Company	2010–2020	12	https://benjamins.com/catalog/ps	Language use and social normativity, such as in education, political discourse as well as discriminatory language use.
Perspectives in Pragmatics, Philosophy and Psychology	Switzerland	Springer International Publishing AG	2013–2014, 2016–2020	12	https://www.springer.com/series/11797	Theoretical pragmatics and pragmatics.
International Review of Pragmatics	Netherlands	Brill Academic Publishers	2014–2020	13	https://brill.com/view/journals/irp/irp-overview.xml	Different topics in pragmatics
Lodz Papers in Pragmatics	Germany	Versita (Central European Science Publishers)	2015–2018, 2020	16	https://www.degruyter.com/journal/key/lpp/html	Human communication, both in everyday interactions and in the media, whether verbal or written, institutional or interpersonal.
Current Research in the Semantics/Pragmatics Interface	Netherlands	Brill Academic Publishers	2007–2016, 2018		https://brill.com/view/serial/CRISPI?lang=en	Different topics in semantics and pragmatics
Pragmatics and Beyond New Series	Netherlands	John Benjamins Publishing Company	2018–2020		https://benjamins.com/catalog/pbns	Different topics in pragmatics, linguistic and sociocultural contexts and different theoretical and methodological perspectives.
Studies in Pragmatics	Netherlands	Brill Academic Publishers	2009–2010, 2012, 2014–2017		https://brill.com/view/serial/SIP	Theoretical, analytical, and applied pragmatic studies.
East Asian Pragmatics	United Kingdom	Equinox Publishing Ltd.	2018–2020	6	https://journal.equinoxpub.com/EAP	East Asian language use contributing to pragmatics.

**Table 2:** Search strings for retrieving data on pragmatics from Scopus, WOS and Lens.

Wednesday, 9 March 2022 **Scopus** TITLE-ABS-KEY ({pragmatics}) AND (LIMIT-TO (DOCTYPE, “ar”)) AND (LIMIT-TO (LANGUAGE, “English”)) 6554 document results from 1939 to 2022
**WOS** TS=(“pragmatics”) and Web of Science Core Collection (Database) and **English** (Languages) and Articles or Early Access (Document Types) 6597 results from Web of Science Core Collection from 1966 to February 2022 **WOS** (To get most cited authors and references in CiteSpace) **“pragmatics”** (Topic) and **Articles** (Document Types) and **English** (Languages) and **2022** or **2021** or **2020** or **2019** or **2018** (Publication Years) 2222 results from Web of Science Core Collection from 2018 to February 2022
**Lens** source.title:pragmatics Filters: Publication Type Scholarly Works (11,230) from 1966 to 2022

**Table 3:** Top cited documents of pragmatics based on citation reports from Scopus, WOS and Lens.

No.	Source Title	Citation	Citations by Database		
			Scopus	WOS	Lens
1	A grammar of institutions	[59]	694	X	X
2	A pedagogy of multiliteracies: Designing social futures	[60]	2892	2114	X
3	Action and embodiment within situated human interaction	[61]	X	X	1927
4	Context, Activity and Participation	[62]	X	X	574
5	Development of the Children’s Communication Checklist (CCC): A method for assessing qualitative aspects of communicative impairment in children	[63]	X	390	Χ
6	From communication to language-a psychological perspective	[64]	594	X	X
7	Information Structure in Discourse: Towards an Integrated Formal Theory of Pragmatics	[65]	X	X	719
8	Moment analysis and translanguaging space: discursive construction of identities by multilingual Chinese youth in Britain	[66]	X	X	765
9	Neurophenomenology: A methodological remedy for the hard problem	[67]	821	X	X
10	Ontology mapping: The state of the art	[68]	861	X	X
11	Perspectives on politeness	[69]	X	X	699
12	Pinning down the concept of interface in bilingualism	[70]	X	379	X
13	Politeness phenomena in modern Chinese	[71]	X	X	715
14	Pragmatics of measuring recognition memory-applications to dementia and amnesia	[72]	2437	2437	X
15	Pragmatics, modularity and mind-reading	[73]	X	484	X
16	Qualitative content analysis in nursing research: Concepts, procedures and measures to achieve trustworthiness	[74]	9320	8781	X
17	Reexamination of the universality of face: Politeness phenomena in Japanese	[75]	X	X	653
18	Small wins: Redefining the scale of social problems	[76]	624	X	X
19	Successfully completing case study research: combining rigour, relevance and pragmatism	[77]	X	371	X
20	The discursive accomplishment of normality: On ?€?lingua franca?€? English and conversation analysis	[78]	X	X	768
21	The pragmatics of model-driven development	[79]	841	555	X
22	Towards an anatomy of impoliteness	[80]	X	X	859
23	What are discourse markers	[81]	X	Χ	745
24	When children are more logical than adults: experimental investigations of scalar implicature	[82]	X	388	X
25	Wisdom: A metaheuristic (pragmatic) to orchestrate mind and virtue toward excellence	[83]	715	571	X

**Table 4:** Major pragmatics clusters.

Cluster ID	Size	Silhouette	Label (LSI)	Label (LLR)	Label (MI)	Average Year
0	154	0.827	pragmatic competence	speech act (741.32, 1.0 × 10^4^)	syntax-discourse interface (3.68)	1997
1	133	0.748	case study	relevance-theoretic approach (542.65, 1.0 × 10^4^)	syntax-discourse interface (2.87)	1994
Scopus						
0	418	0.663	natural language processing	autism spectrum disorder (5841.56, 1.0 × 10^4^)	character equivalence (6.1)	2004
1	297	0.472	autism spectrum disorder	pragmatic disorder (1561.88, 1.0 × 10^4^)	character equivalence (0.87)	1991
2	212	0.785	autism spectrum disorder	pragmatic language (3311.06, 1.0 × 10^4^)	character equivalence (1.47)	2001

**Table 5:** Citation counts for pragmatics research.

WoS			Scopus		
Citation	Reference	Cluster ID	Citation	Reference	Cluster ID
231	Grice [84]	3	25	Bambini [90]	0
154	Brown [86]	0	19	Der Van [91]	1
115	Grice [6]	1	19	Haugh [92]	4
100	Leech [19]	0	18	Macagno [93]	93
100	Levinson [94]	1	16	Taguchi [95]	14
100	Sperber [8]	1	15	Bosco [96]	3
96	Austin [85]	0	13	Adams [97]	5
92	Levinson [5]	2	12	Kasher [98]	10
81	Deirdre [8]	1	12	Capone [99]	93
56	Bates [100]	2	11	Kadar [101]	4

**Table 6:** Bursts detection for research on pragmatics.

WoS			Scopus		
Burst	Reference	Cluster ID	Burst	Reference	Cluster ID
4.52	Brown [86]	0	6.33	Haugh [92]	4
3.77	Goffman [102]	0	5.71	Taguchi [95]	14
3.72	Austin [85]	1	4.63	Capone [99]	93
3.45	Silverstein [103]	1	4.24	Verschueren [104]	89
3.44	Harnish [105]	1	3.96	Der Van [91]	1
3.18	Kecskes [106]	0	3.91	Kadar [101]	4
3.18	Wilson [107]	1	3.62	Kasher [98]	10
3.18	Mills [108]	0	3.06	Goodman [109]	11
3.11	Grice [6]	1	2.91	Bott [110]	13
2.92	Bishop [87]	3	2.87	Nerlich [111]	77

**Table 7:** Centrality calculation for top central authors in pragmatics.

WoS			Scopus		
Centrality	Reference	Cluster ID	Centrality	Reference	Cluster ID
314	Grice [84]	3	14	Bambini [90]	0
242	Brown [86]	0	12	Der Van [91]	1
224	Levinson [5]	2	10	Zurif [112]	2
221	Grice [6]	1	9	Bosco [96]	3
221	Levinson [94]	1	8	Arcara [89]	0
219	Sperber [8]	1	8	MacWhinney [113]	2
196	Deirdre [8]	1	7	Katsos [114]	1
175	Austin [85]	0	7	O’Neil-Pirozzi [115]	6
170	Leech [19]	0	7	Ness [115]	6
148	Horn [116]	1	7	Byom [115]	6

**Table 8:** Sigma calculation for top 10 authors in pragmatics.

WoS			Scopus		
Sigma	Reference	Cluster ID	Sigma	Reference	Cluster ID
0	Grice [84]	3	0	Bambini [90]	0
0	Brown [86]	0	0	Der Van [91]	1
0	Levinson [5]	2	0	Zurif [112]	2
0	Grice [6]	1	0	Bosco [96]	3
0	Levinson [94]	1	0	Arcara [89]	0
0	Sperber [8]	1	0	MacWhinney [113]	2
0	Deirdre [8]	1	0	Katsos [114]	1
0	Austin [85]	0	0	O’Neil-Pirozzi [115]	6
0	Leech [19]	0	0	Ness [115]	6
0	Horn [116]	1	0	Byom [115]	6

## Data Availability

The data presented in this study are available on request from the first author.
